# Superconducting Bio-Inspired Au-Nanowire-Based Neurons

**DOI:** 10.3390/nano12101671

**Published:** 2022-05-13

**Authors:** Olga V. Skryabina, Andrey E. Schegolev, Nikolay V. Klenov, Sergey V. Bakurskiy, Andrey G. Shishkin, Stepan V. Sotnichuk, Kirill S. Napolskii, Ivan A. Nazhestkin, Igor I. Soloviev, Mikhail Yu. Kupriyanov, Vasily S. Stolyarov

**Affiliations:** 1 Institute of Solid State Physics RAS, 142432 Chernogolovka, Russia; oskrya@gmail.com; 2 Center for Advanced Mesoscience and Nanotechnology, Moscow Institute of Physics and Technology, 9 Institutskiy per., 141700 Dolgoprudny, Russia; andrey_shishkin@mail.ru (A.G.S.); nazhestkin@phystech.edu (I.A.N.); vasiliy.stoliarov@gmail.com (V.S.S.); 3 Skobeltsyn Institute of Nuclear Physics, Lomonosov Moscow State University, 119991 Moscow, Russia; tanuior@gmail.com (A.E.S.); r3zz@mail.ru (S.V.B.); mkupr@pn.sinp.msu.ru (M.Y.K.); 4 Faculty of Physics, Moscow State University, 119991 Moscow, Russia; nvklenov@gmail.com; 5 Dukhov All-Russia Research Institute of Automatics, 101000 Moscow, Russia; sotnya777@mail.ru; 6 Department of Materials Science, Lomonosov Moscow State University, 119991 Moscow, Russia; napolskiiks@my.msu.ru; 7 Department of Chemistry, Lomonosov Moscow State University, 119991 Moscow, Russia; 8 Russian Quantum Center, 100 Novaya Street, 143025 Skolkovo, Russia; 9 National University of Science and Technology MISIS, 4 Leninsky prosp., 119049 Moscow, Russia

**Keywords:** Josephson effect, superconductivity, artificial neural networks, nanodevices, neuromorphic systems

## Abstract

High-performance modeling of neurophysiological processes is an urgent task that requires new approaches to information processing. In this context, two- and three-junction superconducting quantum interferometers with Josephson weak links based on gold nanowires are fabricated and investigated experimentally. The studied cells are proposed for the implementation of bio-inspired neurons—high-performance, energy-efficient, and compact elements of neuromorphic processor. The operation modes of an advanced artificial neuron capable of generating the burst firing activation patterns are explored theoretically. A comparison with the Izhikevich mathematical model of biological neurons is carried out.

## 1. Introduction

Modeling the processes in the brain of living beings is a complex and urgent task [1,2,3,4,5,6,7,8,9]. One of the main problems in this area is an insufficient number of neurons and synapses in modern Complementary-Metal-Oxide-Semiconductor (CMOS) neuromorphic processors. Their complexity is closely tightened by large power consumption and heat dissipation of the circuits. The best CMOS neuromorphic systems simulate the networks consisting of about 1 million neurons and a quarter of a billion synapses [10,11,12]. However, the most ambitious biological projects state the goals of $10^{10}$ neurons and $10^{14}$ synapses [13]. The demand for such a high complexity makes important the consideration of novel physical principles for signal transmission and processing.

There are many approaches addressing this issue. Among others, the emerging nonvolatile memory devices [14] are pushing the limits beyond CMOS technology. In particular, ferroelectric memory devices with fast write/read times (of an order of 1 ns) have been recognized as promising neuromorphic elements [15]. They are compatible with CMOS circuits operating at the same voltage level, while consuming several orders of magnitude less energy (of an order of fJ) than CMOS neuron composed of dozens of transistors [16].

**Figure 1:**
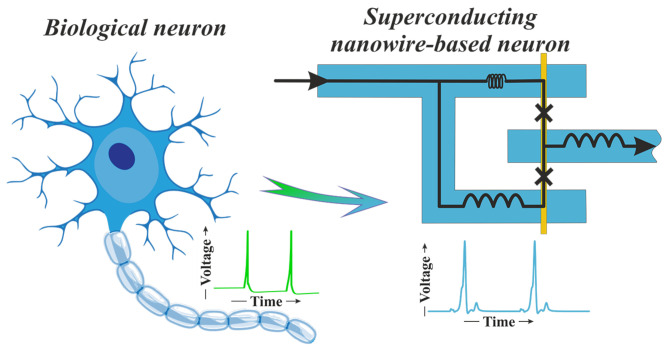
Sketch of a biological neuron and its artificial counterpart made of a superconducting material with a normal metal nanowire.

The application of superconducting materials [17,18,19] also allows for competing CMOS in the implementation of artificial neurons. Josephson junction switching provides the generation of picosecond-width quantized voltage spike of ∼mV amplitude accompanied by sub-aJ energy dissipation [20,21]. The shape of this spike can be quite close to the one produced in neurophysiological processes [22]. It was shown that an artificial neuron can be implemented using only two Josephson junctions [22,23] (see illustration in Figure 1). This is an order of magnitude less than the number of transistors in CMOS counterparts [16]. At the same time, it is possible to implement the synaptic memory using just single junction with ferromagnetic material [24,25,26,27,28,29,30]. The superconductivity enables ballistic signal transfer along the wires. The voltage spikes can be transmitted with a speed approaching the speed of light over long distances with low crosstalk [31]. Note that the Josephson junctions can be fabricated in any layer of multilayer superconducting circuit [32]. These features are favor for the implementation of complex and energy-efficient 3D neural networks closely mimic biological nerve tissue.

While the superconducting neural networks of various kinds are rapidly developed currently [22,23,24,28,33,34,35,36,37,38,39,40], their complexity is severely limited by the low integration density of superconducting circuits [41,42]. One of the main reasons for this is a comparatively large area (an order of a micron to few tenths of a micron squared) of commonly used superconductor-insulator-superconductor tunnel Josephson junction [32]. Elaboration of nanoscale junctions are highly important for many applications [20,43,44,45,46,47,48,49,50,51,52]. In this work, we develop the nanowire-based Josephson structures for the implementation of superconducting bio-inspired neurons. We further elaborate the superconducting artificial neuron schematic enabling operation modes corresponding to important biological activity missed in previously proposed devices.

## 2. Results and Discussion

### 2.1. Preparation of Samples and Experimental Results

The fabricated samples are based on gold nanowires, 60 nm in diameter. The nanowires were grown by metal electrodeposition into porous templates of anodic alumina from cyanide-free Ecomet 04-ZG electrolyte solution at a constant deposition potential of −1.0 V versus Ag/AgCl reference electrode. Different types of superconducting quantum interferometers (SQUIDs) were fabricated using e-beam lithography and magnetron sputtering of superconducting niobium (see Supplementary Materials and Ref. [53] for more details).

**Figure 2:**
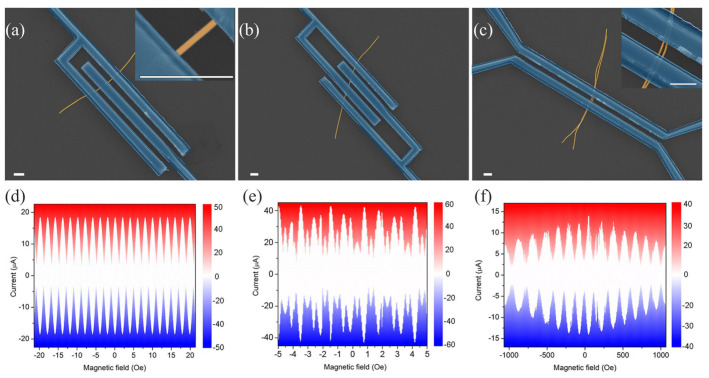
Scanning electron microscope images of (**a**) *sample A* (insert—Zoom of the Josephson junction), (**b**) *sample B*, and (**c**) *sample C* with zoom of the central part of the *sample C*. The scale bar is 1 μm. The corresponding sets of current-voltage characteristics in various magnetic fields for these samples are shown in panels (**d**–**f**); voltage scale is in μV (displayed in color).

We have implemented two types of SQUID cells. The first one is a device with two Josephson weak links, *sample A*, shown in Figure 2a with a quantization loop area of 5.1 μm${}^{2}$. The other one is a SQUID cell with three Josephson junctions, *sample B* (Figure 2b), with two loops having quantization areas of 11.3 and 13.8 μm${}^{2}$. We have also implemented a more exotic variant of a two-junction SQUID composed of closely spaced nanowires placed between bulk superconducting electrodes—*Sample C* (Figure 2c). Here the working area between two wires is about 0.5 μm${}^{2}$.

The *sample A* serves as a reference device demonstrating operation of conventional SQUID fabricated using the proposed technology. At the same time, the SQUID is a basic cell for previously proposed superconducting bio-inspired neuron [22]. The *sample B* is a prototype cell of an improved artificial neuron capable of burst firing (or *bursting*) corresponding to specific activation patterns of biological neurons in the central nervous system and spinal cord, see below. Finally, with the *sample C* we test an approach for a possible miniaturization of SQUIDs. The area of this device is so small that we see the characteristic Fraunhofer pattern in just a dozen periods of typical oscillations of critical current-magnetic field dependencies.

The current–voltage characteristics of the samples were measured in a dilution refrigerator. They exhibit some asymmetry in their critical current which can be attributed to a frozen magnetic flux (see Supplementary Materials). The shape of the I–V curves becomes hysteretic below 1 K temperature that is likely due to thermal effects caused by the difference between the physical and electronic temperatures [54,55,56]. The critical current of Josephson junctions decreases by about an order with the temperature increase from 20 mK to 2.4 K, see Supplementary Materials.

The sets of I-V curves presented in Figure 2d–f were measured at T=20 mK in various perpendicular magnetic fields. The *sample A* with a maximum critical current, $\max(I_{CA})$, of 18.5 μA, shows regular oscillations of critical current-magnetic field dependence with a period of about H=2 Oe (Figure 2d). The symmetry of $I_{CA}\left(H\right)$ curve shape indicates that the critical currents of the SQUID Josephson junctions are close in values. The depth of the critical current modulation can be used for the estimation of the SQUID circuit inductance to be less than $\Phi_{0}/2\pi I_{CA}$, where $\Phi_{0}=h/2e$ is the flux quantum (*h* is Planck’s constant and *e* is the electron charge). Similar oscillations of $I_{CB}\left(H\right)$ for the *sample B* is irregular due to more complex geometry of the cell (the maximum critical current is $\max(I_{CB})=42.5\;\mu$A, see Figure 2e). For the *sample C*, the period of the oscillations is much longer and is approximately 140 Oe. The maximum critical current, $\max(I_{CC})=14$ μA, decays twice in a field of 1000 Oe (Figure 2f).

On the one hand, the experimental data show that the fabricated Josephson junctions possess ∼μA critical current and normal state resistance of several Ohms exhibiting no overheating above 1 K temperature. The critical current can be further improved by shrinking the gap between superconducting electrodes. On the other hand, these junctions can be connected in SQUID cells such that even the cell with closely located nanowires provides the typical magnetic flux-voltage characteristics. These results have motivated us to investigate the bio-inspired neurons which can be implemented utilizing nanowire–Josephson–junction SQUID-based cells.

### 2.2. Bio-Inspired Neuron

**Figure 3:**
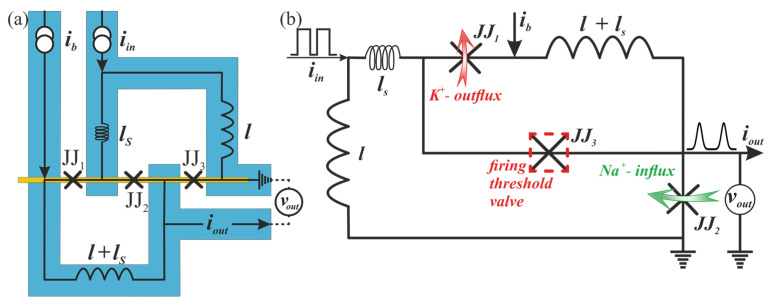
(**a**) Sketch of the proposed superconducting bio-inspired neuron with nanowire-based Josephson junctions. (**b**) Schematic of the proposed bio-inspired neuron.

A conventional SQUID cell such as *sample A* can be naturally used for the implementation of the SQUID-based neuron proposed in [22]. However, more useful circuit may be developed using three-Josephson-junction cell corresponding to the *sample B*, see Figure 3 and Supplementary Materials. This circuit can be analyzed using the resistively shunted junction model with capacitance of Josephson junctions [20,21], while the experimentally studied junctions have vanishing self capacitance, here we include it into consideration to capture all possible operation regimes of the considered neuron circuit. We assume that the critical currents of the junctions $JJ_{1}$ and $JJ_{2}\;$ (Figure 3) are equal to $I_{C0}$ and fixed, whereas the critical current of $JJ_{3}\;$, $\;I_{C3}\;$, can be varied. Kirchhoff’s equations augmented by the phase-based circuit Equations [57] lead to the following system (see Supplementary Materials):

(1) $$\left\{\begin{array}{c} \ddot{\phi_{1}}=-\frac{1}{\beta }\left\{i_{b}+\lambda \left(\phi_{1}-\phi_{3}\right)+\dot{\phi_{1}}+\sin\phi_{1}\right\},\\ \ddot{\phi_{2}}=\frac{1}{\beta }\left\{i_{b}+i_{in}\Lambda -\lambda \left(\phi_{2}+\phi_{3}\right)-\dot{\phi_{2}}-\sin\phi_{2}\right\},\\ \ddot{\phi_{3}}=\frac{1}{\beta }\left\{\frac{1}{\eta }\left(i_{b}+i_{in}\Lambda +\lambda \left(\phi_{1}-\phi_{2}-2\phi_{3}\right)\right)-\dot{\phi_{3}}-\sin\phi_{3}\right\}, \end{array}\right.$$

where input current, $i_{in}$, and bias current, $i_{b}$, are normalized to the reference current $I_{C0}$, whereas η is the ratio of $I_{C3}$ to $I_{C0}$. Λ=λl, $\lambda ={\left(l+l_{S}\right)}^{-1}$, inductances, *l*, $l_{s}$, are normalized to $\Phi_{0}/2\pi I_{C0}$. $\phi_{1,2,3}$ are Josephson phases of the junctions, dots indicate derivatives (the number of dots means the derivative order) with respect to time, *t*, normalized to the inverse plasma frequency, $\tau =t\omega_{p}$, $\omega_{p}=\sqrt{2\pi I_{C0}/\Phi_{0}C}$, where *C* is the junction capacitance. $\beta =\omega_{c}RC$ is the Stewart–McCumber parameter, *R* is the junction resistance in the normal state, and $\omega_{c}=2\pi I_{C0}R/\Phi_{0}$ is the characteristic frequency.

The circuit dynamics are governed mainly by two parameters at fixed bias current value. They are the normalized critical current of the third junction, η, determining the neuron firing threshold, and the normalized capacitance of the junctions, β, responsible for the refractory period.

**Figure 4:**
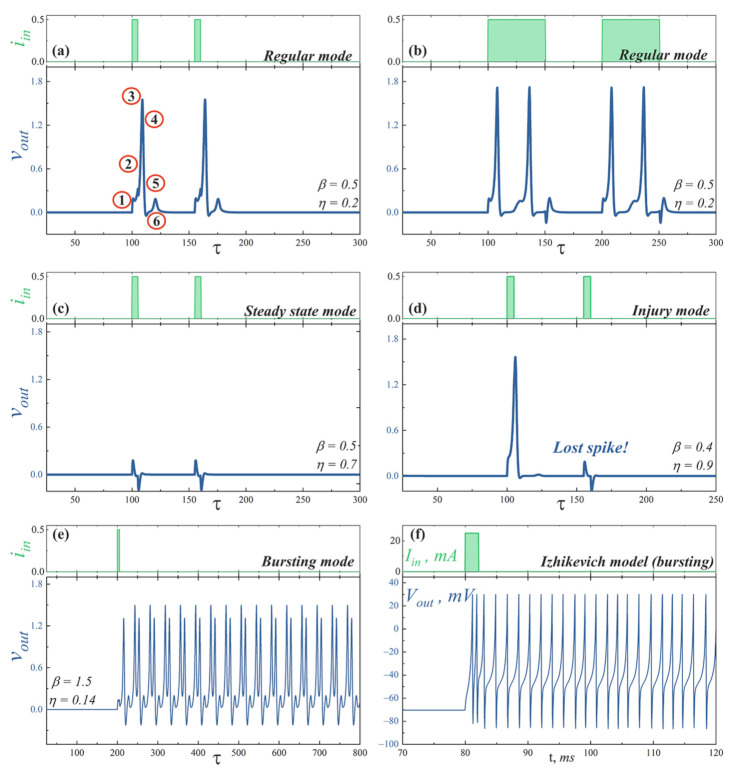
Simulations of the superconducting bio-inspired neuron dynamics in the various operation modes: (**a**,**b**) *regular mode* (β=0.5, η=0.2), (**c**) *steady state mode* (β=0.5, η=0.7), (**d**) *injury mode* (β=0.4, η=0.9), and (**e**) *bursting mode* (β=1.5, η=0.14). The figures show the output voltage across the second Josephson junction of the cell stimulated by the input current pulse. The circuit parameters are $i_{b}=1.9$, l=5, $l_{S}=3.85$. (**f**) Bursting dynamics obtained in the framework of the Izhikevich model, see Figure S3d of Supplementary Materials and its description therein.

The first and second Josephson junctions play the role of sodium ($Na^{+}$) and potassium ($K^{+}$) ion channels in the neuron membrane [1,58], respectively. The voltage across the cell, $v_{out}$ (see Figure 3b), reflects the following processes during the neuron firing, see the circled numbers on a spike shown in Figure 4a: 1—the neuron is stimulated above the firing threshold, $Na^{+}$ channels are opened so that $Na^{+}$ begins to enter the cell; 2—$K^{+}$ channels are opened, $K^{+}$ begins to leave the cell; 3—$Na^{+}$ channels become refractory, no more $Na^{+}$ enters the cell; 4—$K^{+}$ leaves the cell and rapidly repolarizes the membrane; 5—$K^{+}$ channels are closed and $Na^{+}$ channels reset; 6—extra $K^{+}$ outside diffuses away.

The proposed neuron is capable of mimic the biological activity corresponding to the modes presented in Figure 4 (see Supplementary Materials for comparison with simulations performed within the framework of the Izhikevich mathematical model of biological neurons):

- ***Regular mode*** shows the typical response of a neuron to external stimulation. A short input current pulse of a sufficient amplitude causes single spike, whereafter the system returns to a stable state, see Figure 4a. A long pulse leads to repeated overcoming of the firing threshold. Thus, a series of spikes is observed, Figure 4b. The interspike interval is determined by a neuron refractory period, which, in consequence, is related to the recovery of $Na^{+}$ channels.
- ***Steady state mode*** (Figure 4c) is characterized by the weak damped output pulses. This is analog of the maintenance of constant internal concentrations of ions in the cell in response to an under threshold stimulation.
- ***Injury mode*** (Figure 4d) is characterized by the losses of spikes, or vice versa, the generation of “extra” spikes. This mimics the biophysical abnormality caused by different nervous diseases and neuron injuries.
- ***Bursting mode*** (Figure 4e) demonstrates the generation of a series of spikes in response to singe stimulating current pulse. Such behavior may be the result of the complex neuron interaction in the network. However, this can also be a consequence of internal processes in a neuron. In the last case, the reason is the after-depolarization (ADP), a membrane depolarisation at the last stages of repolarisation (circled “4” on the spike shown in Figure 4a) [59,60]. A slow sodium current appears at membrane voltage ∼−50*…*−70 mV and overcomes outward $K^{+}$ current, causing a membrane voltage to rise again. Such current is resistant to inactivation and may last for long times. The bursting pattern parameters—the spike sequence frequency and its length—are determined by the concentration of ion channels of different kinds, properties of these channels, and ionic concentrations in extracellular space. Though only relatively small cohort of neurons in vivo exhibits a bursting behavior [61,62,63], it plays an important role in synaptic plasticity [64,65], synchronization of big neuron groups [66], detection of frequency features of input stimuli [67], information encoding [68,69], and reliability of synaptic transmission [64,70], which may be crucial for processing of important stimuli [71].
- Figure 4f illustrates the bursting dynamics of biological neuron simulated in the frame of the fractional-order Izhikevich model [72,73] for comparison, see also Supplementary Materials.

**Figure 5:**
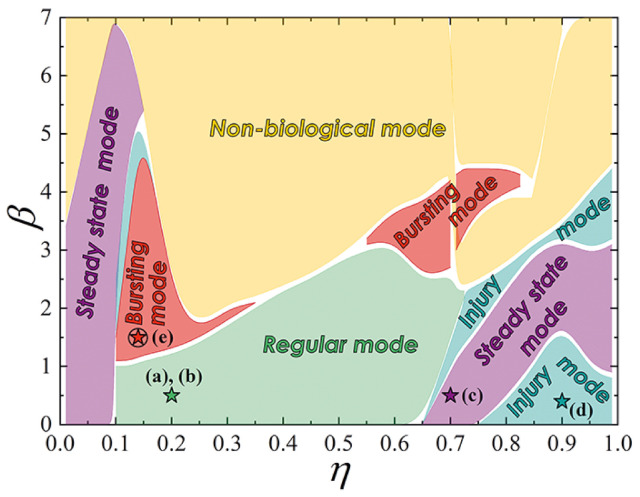
Map of the proposed bio-inspired neuron operating modes on the plane of parameters (β, η). White boundaries represent the areas where transient processes complicating identification of a particular mode take place. Stars mark parameters taken for simulations presented in Figure 4. The regular, steady state, and injury modes can be obtained in the overdamped circuits (β⟶0) such as the studied experimental samples. Implementation of the bursting mode requires underdamped system.

The map of the proposed neuron operation modes on the plane of parameters (η, β) is shown in Figure 5. The parameters used for simulations presented in Figure 4 are marked by stars.

The non-biological dynamics, which are not observed in vivo under any conditions, take place at small damping in the system (large β). In the overdamped circuits (β⟶0) such as the studied experimental samples, the *steady state*, *regular*, and *injury modes* can be obtained. Small critical current of the third Josephson junction (η≈0) effectively blocks the inflow of the input current into the output junction, $JJ_{2}$, redirecting it to the inductance, *l*, connected to the ground, see Figure 3b. This results in the *steady state mode* where no spike is produced. An increase in η value opens access to the output junction, such that the input current pulse triggers successive switching of the junctions $JJ_{2}$, $JJ_{3}$, $JJ_{1}$. This mimics $Na^{+}$ ions inflow succeeded by $K^{+}$ outflow corresponding to the *regular mode*. At larger η values the neuron circuit becomes bistable with one enabling and one inhibiting intracellular current configuration for the output junction switching. In this case, the *steady state* and *injury modes* are determined by the initial current configuration in the circuit: inhibiting or enabling (switched to inhibiting after the first spike), respectively.

Unfortunately, the *bursting mode* clearly requires finite capacitance to maintain periodic switching of the Josephson junctions. This indicates the necessity for complication of the studied cell circuit, e.g., an introduction of the shunting capacitor.

While the *regular* and *steady state mode* can be reproduced in the previously proposed bio-inspired neuron [22], the *injury* and *bursting modes* appear only in the presented advanced neuron cell.

## 3. Conclusions

The distinctive features of the superconducting technology are the high frequency operation and high energy efficiency. The possibility of processing of up to $10^{10}$ spikes/s by an artificial neuron (versus maximum ∼453 spikes/s in the human brain [74] or 100–200 spikes/s widely spread [75,76]) and power consumption of about 0.1 MW [28] for human brain model pave the way for high performance neurophysiological simulations. We have addressed the low integration density issue of superconducting circuits by the development of technology intended for fabrication of compact SQUIDs utilizing nanowire-based nanobridge Josephson junctions. We have experimentally demonstrated two types of SQUIDs suitable for the implementation of bio-inspired neurons. Numerical simulations show that the proposed three-junction cell is capable of mimic specific biological neuron activity missed in previously presented superconducting artificial neurons. In this mode of operation, regular and irregular spike sequences are generated as activation patterns occurring in vivo in many cases including stereotypical motor programs, neural coding, and neuropathologies. An introduction of capacitance in the cell designs and studying options for signal transfer between the artificial neurons are urgent tasks in the considered research direction.

## Data Availability

The data presented in this study are available on request from the corresponding author.

## Supplementary Materials

The following supporting information can be downloaded at: https://www.mdpi.com/article/10.3390/nano12101671/s1. Figure S1: I-V characteristics of samples A (a), B (b), and C (c) measured at different temperatures at zero magnetic field. Figure S2: Sketch with superimposed schematic of the sample B (a) and a bio-inspired neuron cell based on it (b). (c) The lumped element circuit of the neuron cell. Figure S3: Numerical simulations of biological neurons in the framework of the the fractional-order Izhikevich model. The model parameters are as follows. (a), (b) regular mode: a = 0:02, b = 0:2, c = −65, d = 8, a = 1, (c) injury mode: a = 2 · 10${}^{-5}$, b = 0:2, c = −65, d = 8, a = 1, (d) bursting mode: a = 0:02, b = 0:2, c = −65, d = 8, a = 0:63, (e) bursting with early termination: a = 0:1, b = 0:2, c = −65, d = 2, a = 0:65, (f) regular mode (bursting is not observed): a = 0:1, b = 0:2, c = −65, d = 2, a = 0:7. References [73,74,77,78,79,80,81,82] are cited in the supplementary materials.
