# Supplementary material for: Superconducting Bio-Inspired Au-Nanowire-Based Neurons
Source: Nanomaterials (Basel). 2022 May 13;12(10):1671. doi: 10.3390/nano12101671 (PMC9147065; doi:10.3390/nano12101671)
Supplement: Supplementary file 1 [file nanomaterials-12-01671-s001.zip › nanomaterials-1690232-supplementary.pdf]

Superconducting Bio-inspired Au-Nanowire-Based Neurons Supplementary Materials

Olga V. Skryabina, Andrey E. Schegolev, Nikolay V. Klenov, Sergey V. Bakurskiy, Andrey G. Shishkin, Stepan V. Sotnichuk, Kirill. S. Napolskii, Ivan A. Nazhestkin, Igor I. Soloviev, Mikhail Yu. Kupriyanov, Vasiliy S. Stolyarov

Justification of the chosen method for experimental sample fabrication

The templated electrodeposition of metal nanowires allows one to fabricate single-crystal anisotropic nanostructures. Their utilization for the implementation of Josephson junctions results in compact devices possessing large enough critical currents despite of relatively wide gap between the electrodes. In combination with the comparative simplicity of the technology, this is the reason for choosing this approach for the first stage of development of neuromorphic basic elements. The obtained experimental results together with theoretical studies of bio-inspired neuron cells are planned to be a guidance for the further transfer to more complex technology of planar nanobridge Josephson junctions considered in Ref. [1], which is suitable for very-large scale integration.

Electrochemical preparation of Au nanowires

Gold nanowires were fabricated by a templated electrodeposition technique using porous anodic aluminum oxide (AAO) as a template. AAO film was made by two-stage anodization of high-purity (99.99%) aluminum in 0.3 M $H_2C_2O_4$ at 40 V. The electrolyte was rigorously agitated, and its temperature was kept constant at 0 °C. The anodization process was ceased when the AAO thickness reached 35 μm. The remaining aluminum was dissolved in a mixture of Br_2 and CH_3OH (1:10 vol.). The barrier layer was etched away in 3 M H_3PO_4 with controlling the moment of pore opening and their further widening to a diameter of 60 nm. At the last stage of template preparation, a 240-nm-thick gold layer was deposited on the bottom side of the AAO using magnetron sputtering to create an electrical contact. Then gold nanowires were electrodeposited at room temperature from cyanide-free Ecomet 04-ZG electrolyte solution at a constant deposition potential of –1.0 V versus Ag/AgCl reference electrode. A pulse of –1.2 V with a duration of 0.1 s was set before the deposition to increase the nucleation rate. During the experiment, the electrolyte was constantly agitated using a magnetic stirrer at a rate of 400 rpm. The extraction of gold nanowires from the AAO template was carried out in 1.25 M NaOH solution. Finally, the nanowires were washed repeatedly with water and isopropanol.

Electron transport measurements

Figure S1 presents the current–voltage characteristics of the samples shown in Fig. 1 of the main text. The asymmetry of the critical currents, especially noticeable in Fig. S1a, is due to the presence of a frozen magnetic flux in the structure. The hysteresis associated with the overheating phenomena decreases with the temperature growth, due to temperature dependence of the superconducting gap.

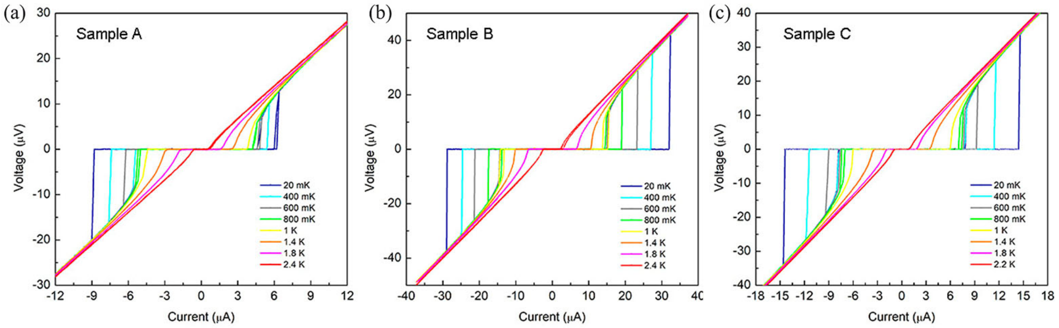

Figure S1: I-V characteristics of samples A (a), B (b), and C (c) measured at different temperatures at zero magnetic field.

Bio-inspired neuron based on the *sample B*

Figure S2a presents a sketch and schematic representation of the *sample B*. “Long” (l) and “short” (l_S) inductors are introduced in the circuit model due to the asymmetry of the superconducting inductive shoulders. External magnetic flux penetrates unequally into the quantization loops of the structure, due to their different areas. This can be taken into account by using two generators of the superconducting phase difference, $\varphi_{1,2}$.

The considered circuit (Fig. S2a) can be simply converted into the bio-inspired neuron cell by the geometry modification presented in Fig. S2b. An equivalent lumped element circuit of the obtained cell (Fig. S2b) is shown in Fig. S2c (Figures S2b,c

correspond to Fig. 2 in the main text). In our analysis of the studied circuit, we normalized all currents to the reference current, I_{C0}, whereas inductances were normalized to the Josephson inductance, $L_J = \Phi_0/2\pi I_{C0}$, where $\Phi_0 = h/2e$ is the flux quantum, h is Planck's constant and e is the electron charge. The Josephson junctions were considered in the frame of the resistively shunted junction model with capacitance [2, 3]:

$$i_{JJ} = \beta\ddot{\phi} + \dot{\phi} + i_{C0}\cdot\sin\phi, \tag{S1}$$

where ϕ is the Josephson phase, i_{JJ} is current flowing through the Josephson junction, i_{C0} is the junction critical current, dots indicate derivatives with respect to time, t, normalized to the inverse plasma frequency, $\tau = t\omega_p$, $\omega_p = \sqrt{2\pi I_{C0}/\Phi_0 C}$, where C is the junction capacitance, $\beta = \omega_c RC$ is the Stewart–McCumber parameter, R is the junction resistance in the normal state, and $\omega_c = 2\pi I_{C0}R/\Phi_0$ is the characteristic frequency, while the geometry of the fabricated Josephson junctions does not imply a capacitance, we include it into consideration to capture all possible operation regimes of the circuit. This capacitance can be further introduced with a shunting capacitor in the cell.

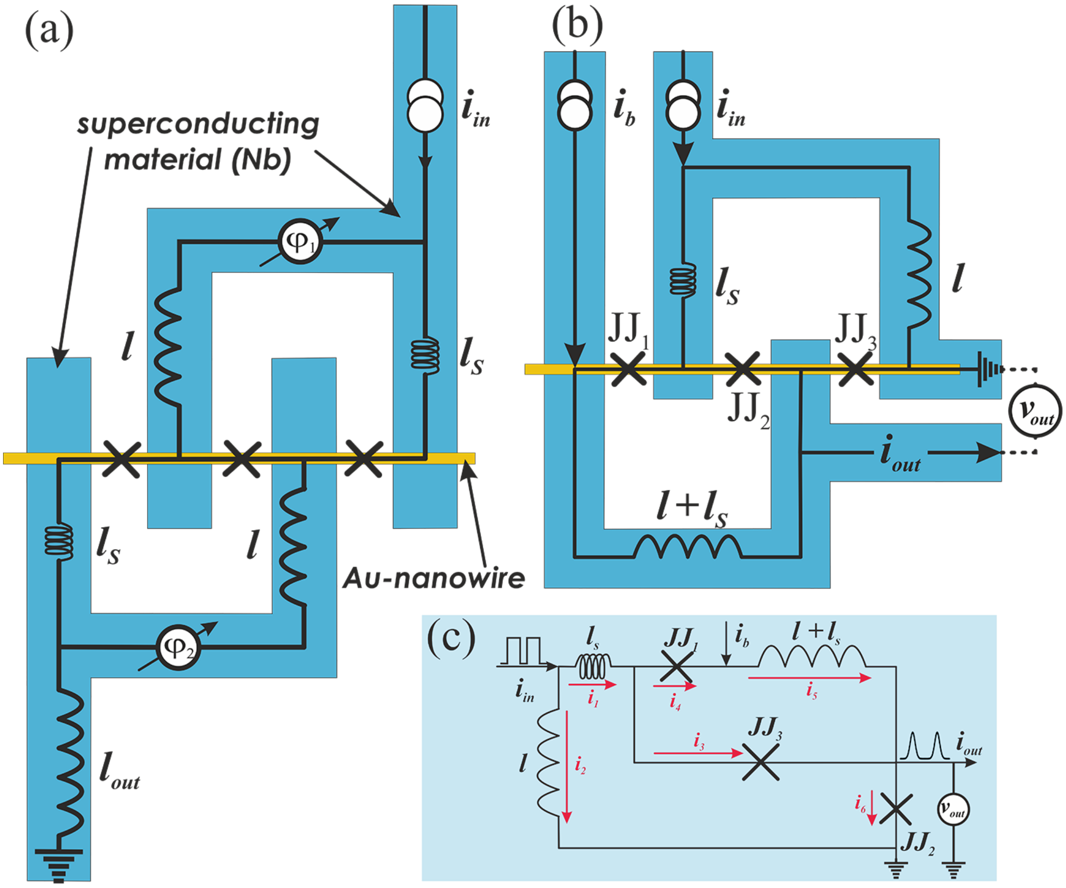

Figure S2: Sketch with superimposed schematic of the *sample B* (a) and a bio-inspired neuron cell based on it (b). (c) The lumped element circuit of the neuron cell.

Kirchhoff's equations for the currents $i_{1,...,6}$, i_{in}, i_b in the considered circuit (Fig. S2c) are as follows:

$$\begin{cases} i_{in} = i_1 + i_2,\ i_1 = i_3 + i_4, \\ i_5 = i_b + i_4,\ i_6 = i_3 + i_5. \end{cases} \tag{S2}$$

The phase-based circuit Equations [4] read

$$\begin{cases} i_2 l = i_1 l_S + \phi_1 + i_5(l + l_S) + \phi_2, \\ i_2 l = i_1 l_S + \phi_2 + \phi_3, \end{cases} \tag{S3}$$

where l, l_s are inductances, see Fig. S2c.

We obtained a system of equations presented in the main text by combining (S1), (S2), (S3):

$$\begin{cases} \ddot{\phi_1} = -\frac{1}{\beta}\left\{i_b + \lambda(\phi_1 - \phi_3) + \dot{\phi_1} + \sin\phi_1\right\}, \\ \ddot{\phi_2} = \frac{1}{\beta}\left\{i_b + i_{in}\Lambda - \lambda(\phi_2 + \phi_3) - \dot{\phi_2} - \sin\phi_2\right\}, \\ \ddot{\phi_3} = \frac{1}{\beta}\left\{\frac{1}{\eta}(i_b + i_{in}\Lambda + \lambda(\phi_1 - \phi_2 - 2\phi_3)) - \dot{\phi_3} - \sin\phi_3\right\}, \end{cases} \tag{S4}$$

where $\Lambda = \lambda l$, $\lambda = (l + l_S)^{-1}$, and η is normalized critical current of the third Josephson junction.

Simulations of biological neuron in the frame of the Izhikevich neuron model

We used the well known Izhikevich neuron model [5] for validation of the results obtained in simulations of the proposed superconducting bio-inspired neuron. The voltage dynamics is governed in this model by fast and slow variables, v and u, as follows:

$$\begin{cases} \dot{v} = 0.04v^2 + 5v + 140 - u + I, \\ \dot{u} = a(bv - u), \end{cases} \tag{S5}$$

for subthreshold voltages, and

$$\begin{cases} \dot{v} \leftarrow c, \\ \dot{u} \leftarrow u + d, \end{cases} \tag{S6}$$

for membrane voltage exceeding 30 mV. In the systems (S5), (S6), a set the recovery timescale, b governs subthreshold oscillations of the membrane potential, c determines the resting membrane potential which is achieved by K^+ currents at the repolarization phase of a spike, and d describes the reset of a neuron after a spike. Different combinations of these parameters correspond to the most known neuron classes in the neocortex [6].

We performed simulations of biological neuron dynamics in Python using the Euler method. The input current amplitude was $I_{max} = 25\ mA$ and the integration time step was $\Delta t = 0.05\ ms$.

The *regular mode* was obtained using the model with parameters: $a = 0.02$, $b = 0.2$, $c = -65$, and $d = 8$. The corresponding dynamics is typical for *regularly spiking* neuron class, excitatory neurons in the brain cortex. After a short stimulus, such neuron generates single spike [6] (after-depolarization (ADP) and after-hyperpolarization (AHP) compensate each other so that no spikes are generated after the first one), see Fig. S3a. Long-lasting input current yields a series of spikes until the current is turned off, see Fig. S3b. *Regularly spiking* neurons exhibit a decrement of spiking frequency during stimulation, a so called adaptation. The interspike intervals are determined by a recovery of sodium conductance. In compliance, the model neuron generates smaller number of spikes within the second stimulating pulse.

The *steady state mode* corresponds to the case where the stimulus is under excitation threshold. In vivo this may result from synaptic plasticity where the threshold is altered.

The *injury mode* was obtained with parameters: $a = 2 \cdot 10^{-5}$, $b = 0.2$, $c = -65$, and $d = 8$, which correspond to the very slow recovery dynamics including spike losses, see Fig. S2c. Such neuron behavior does not correspond to any neuron classes in the brain cortex, and indicates biophysical abnormality. For example, some drugs used in therapy of epilepsy, such as phenytonin, can prolong the refractory period due to the inhibition of voltage-activated sodium channels. This procedure is useful to stop the high-frequency neuron spiking during epileptic seizure [7]. Another reason for the considered mode may be the anomalous increase in opening probability of Ca^{2+} channels. They activate the inward K^+ currents causing additional depolarization. Such effect can be triggered, e.g., by ethanol [8]. Additional depolarization was also observed in hypoxia [9]. Thus, the *injury mode* is suitable for simulations of different nervous diseases and neuron injuries.

The generalization of derivatives to a fractional order [10] allows considering the richer dynamics like a burst firing within the framework of the Izhikevich model [11]. This generalization are widely used in various fields of biology including the modeling of mechanics of respiratory systems, diffusion of drugs, electrical conductivity in tissues etc., see Ref. [12].

The differential equations of the Izhikevich model with derivatives of an order α have the form:

$$\begin{cases} \frac{d^\alpha v}{dt^\alpha} = 0.04v^2 + 5v + 140 - u(t) + I, \\ \frac{d^\alpha u}{dt^\alpha} = a(bv(t) - u(t)). \end{cases} \tag{S7}$$

A fractional-order derivative can be calculated using Caputo's definition [13]:

$$\frac{d^\alpha f(x)}{dt^\alpha} = \frac{1}{\Gamma(1-\alpha)} \int_0^t \frac{f'(u)}{(x-u)^\alpha} du \tag{S8}$$

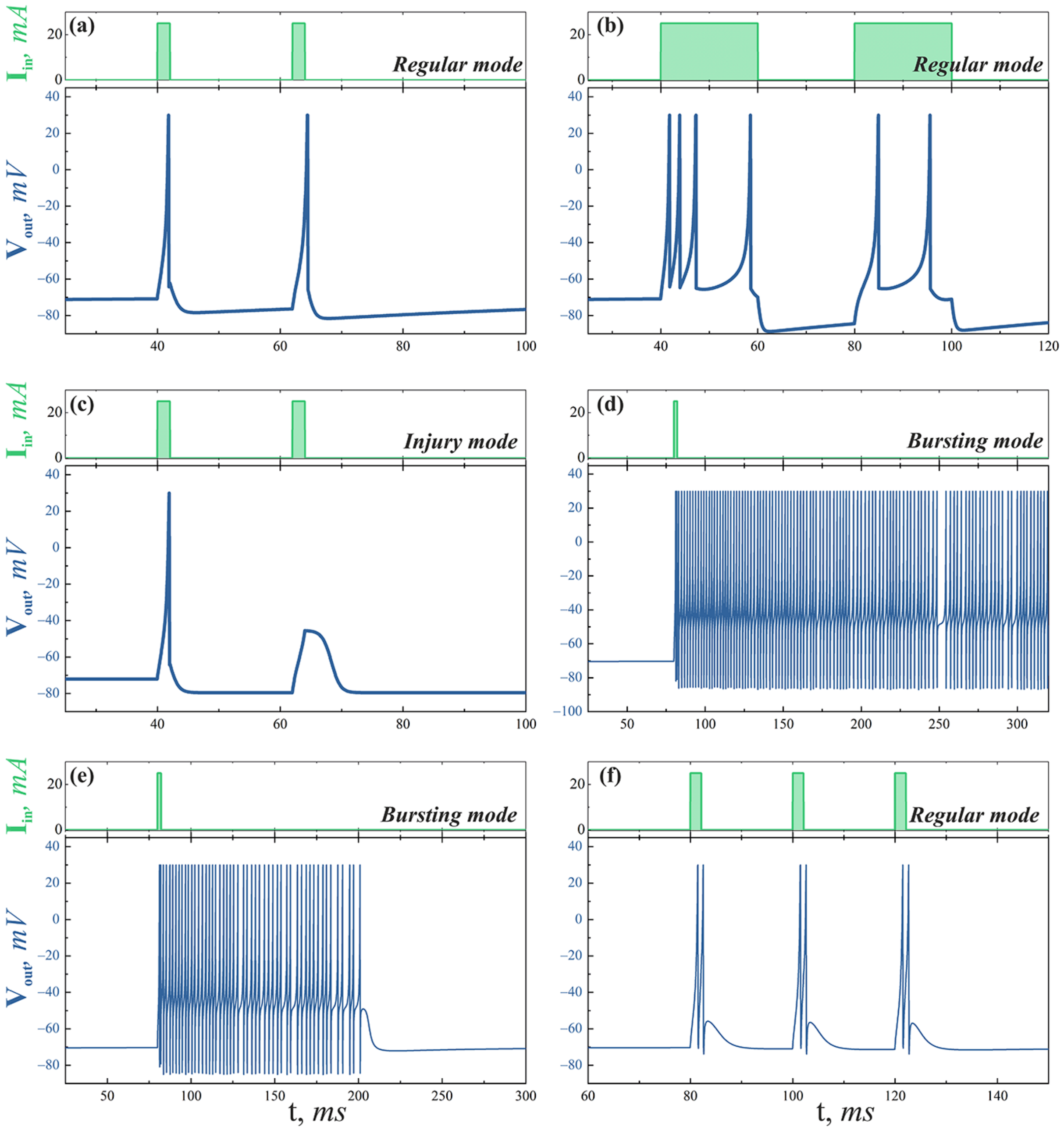

Figure S3: Numerical simulations of biological neurons in the framework of the the fractional-order Izhikevich model. The model parameters are as follows. (a), (b) *regular mode*: $a = 0.02$, $b = 0.2$, $c = -65$, $d = 8$, $\alpha = 1$, (c) *injury mode*: $a = 2{\cdot}10^{-}5$, $b = 0.2$, $c = -65$, $d = 8$, $\alpha = 1$, (d) *bursting mode*: $a = 0.02$, $b = 0.2$, $c = -65$, $d = 8$, $\alpha = 0.63$, (e) bursting with early termination: $a = 0.1$, $b = 0.2$, $c = -65$, $d = 2$, $\alpha = 0.65$, (f) *regular mode* (bursting is not observed): $a = 0.1$, $b = 0.2$, $c = -65$, $d = 2$, $\alpha = 0.7$.

with the further numerical approximation

$$\frac{d^{\alpha} f(x)}{dt^{\alpha}} \approx \frac{(dt)^{-\alpha}}{\Gamma(2-\alpha)} \sum_{k=0}^{N-1} \left[[f(x_{k+1}) - f(x_k)][(N-k)^{1-\alpha} - (N-1-k)^{1-\alpha}] \right], \tag{S9}$$

where Γ is the gamma function, $f(x_i)$ is the history of the f function values, and N is the size of this history.

Finally, the following system can be obtained for each time step of integration of the Izhikevich model:

$$\begin{cases} v(t_N) = dt^{\alpha}\Gamma(2-\alpha)\left[0.04v^2+5v+140-u(t_{N-1})+I\right]+v(t_{N-1})-\sum_{k=0}^{N-2}\left[\left[v(t_{k+1})-v(t_k)\right]\left[(N-k)^{1-\alpha}-(N-1-k)^{1-\alpha}\right]\right], \\ u(t_N) = dt^{\alpha}\Gamma(2-\alpha)\left[a(bv(t_{N-1})-u(t_{N-1}))\right]+u(t_{N-1})-\sum_{k=0}^{N-2}\left[\left[u(t_{k+1})-u(t_k)\right]\left[(N-k)^{1-\alpha}-(N-1-k)^{1-\alpha}\right]\right]. \end{cases} \tag{S10}$$

The *bursting mode* was observed in the fractional-order model with $a = 0.1$, $b = 0.2$, $c = -65$, $d = 2$, and the derivative order parameter $\alpha = 0.63$, see Fig. S3d. This behavior corresponds to *fast-spiking* neurons in the known classification [6]. Such kind of neurons is distinguished by more rapid recovery yielding to higher spiking frequency. Note that the *regular-spiking* neurons unable to produce the burst firing after the end of a stimulus at any values of α.

The bursting was also obtained at $\alpha = 0.65$, see Fig. S3e. However, it was terminated early lasting only ~ 120 ms after the stimulus onset. For the higher values of $\alpha \geq 0.7$, the bursting was not observed, while the neuron showed the dynamics of the regular mode, see Fig. S3f.

[1] I. Soloviev, S. Bakurskiy, V. Ruzhickiy, N. Klenov, M. Y. Kupriyanov, A. Golubov, O. Skryabina, and V. Stolyarov, Physical Review Applied **16**, 044060 (2021).

[2] K. K. Likharev, *Dynamics of Josephson junctions and circuits* (Routledge, 2022).

[3] A. Barone and G. Paterno, *Physics and applications of the Josephson effect*, vol. 1 (Wiley Online Library, 1982).

[4] L. Schindler and C. J. Fourie, IEEE Transactions on Applied Superconductivity (2022).

[5] E. M. Izhikevich, IEEE Transactions on Neural Networks **14**, 1569 (2003).

[6] B. W. Connors and M. J. Gutnick, Trends in Neurosciences **13**, 99 (1990).

[7] B. Wang, W. Ke, J. Guang, G. Chen, L. Yin, S. Deng, Q. He, Y. Liu, T. He, R. Zheng, et al., Frontiers in cellular neuroscience **10**, 239 (2016).

[8] M. Gruß, M. Henrich, P. König, G. Hempelmann, W. Vogel, and A. Scholz, European Journal of Neuroscience **14**, 1246 (2001).

[9] P. Grafe, H. Bostock, and U. Schneider, The Journal of Physiology **480**, 297 (1994).

[10] K. Oldham and J. Spanier, *The fractional calculus theory and applications of differentiation and integration to arbitrary order* (Elsevier, 1974).

[11] W. W. Teka, R. K. Upadhyay, and A. Mondal, Communications in Nonlinear Science and Numerical Simulation **56**, 161 (2018).

[12] C. Ionescu, A. Lopes, D. Copot, J. T. Machado, and J. H. Bates, Communications in Nonlinear Science and Numerical Simulation **51**, 141 (2017).

[13] M. Caputo, Geophysical Journal International **13**, 529 (1967).
